# Challenges for Alzheimer's Disease Therapy: Insights from Novel Mechanisms Beyond Memory Defects

**DOI:** 10.3389/fnins.2018.00037

**Published:** 2018-02-06

**Authors:** Rudimar L. Frozza, Mychael V. Lourenco, Fernanda G. De Felice

**Affiliations:** ^1^Oswaldo Cruz Institute, Fundação Oswaldo Cruz (FIOCRUZ), Rio de Janeiro, Brazil; ^2^Institute of Medical Biochemistry Leopoldo de Meis, Rio de Janeiro, Brazil; ^3^Institute of Biophysics Carlos Chagas Filho, Federal University of Rio de Janeiro, Rio de Janeiro, Brazil; ^4^Department of Biomedical and Molecular Sciences, Centre for Neuroscience Studies, Queen's University, Kingston, ON, Canada

**Keywords:** Alzheimer's disease, inflammation, metabolic derangements, memory defects, preclinical, therapy

## Abstract

Alzheimer's disease (AD), the most common form of dementia in late life, will become even more prevalent by midcentury, constituting a major global health concern with huge implications for individuals and society. Despite scientific breakthroughs during the past decades that have expanded our knowledge on the cellular and molecular bases of AD, therapies that effectively halt disease progression are still lacking, and focused efforts are needed to address this public health challenge. Because AD is classically recognized as a disease of memory, studies have mainly focused on investigating memory-associated brain defects. However, compelling evidence has indicated that additional brain regions, not classically linked to memory, are also affected in the course of disease. In this review, we outline the current understanding of key pathophysiological mechanisms in AD and their clinical manifestation. We also highlight how considering the complex nature of AD pathogenesis, and exploring repurposed drug approaches can pave the road toward the development of novel therapeutics for AD.

## Introduction

Increasing life expectancy has produced a dramatic rise in the number of cases of age-associated diseases, including dementia. Alzheimer's disease (AD) is the most frequent cause of dementia, accounting for 60–80% of all cases (Prince et al., 2016), and epidemiological studies indicate that AD will become even more incident by midcentury, constituting a major personal and societal tragedy. AD is primarily a condition of late life, roughly doubling in prevalence every 5 years after age 65 (Prince et al., 2013), and affects some 47 million people worldwide (Prince et al., 2013). This number is predicted to increase in the next two decades (Prince et al., 2016). The total cost of dementia was estimated around $818 billion in 2010 and has been projected to hit $1 trillion by 2018 worldwide (Prince et al., 2016). This becomes even more dramatic because nearly 60% of people affected by dementia live in low- and middle-income countries.

AD is a complex disorder. While the vast majority of AD cases are sporadic, affecting individuals older than 60 years, genetic mutations cause a rare (<0.5%) familial form of AD, whose symptoms develop earlier, typically between 30 and 50 years of age (Bateman et al., 2010). Further, there is a marked difference in the incidence of AD between women and men. It is estimated that nearly two-thirds of the patients living with AD are women (Alzheimer's Association, 2017), raising the intriguing suggestion that there are biological mechanisms underlying the higher incidence of AD cases in women that still demand to be investigated.

AD is mainly characterized by progressive cognitive impairment. However, as disease progresses, other debilitating non-cognitive symptoms arise, including impaired sleep and appetite, and neuropsychiatric alterations (e.g., depression and apathy) (Ishii and Iadecola, 2015; Lanctôt et al., 2017). In addition, mounting epidemiological studies have supported a link between metabolic disorders and AD (Ott et al., 1996, 1999; Steen et al., 2005; Matsuzaki et al., 2010; Takeda et al., 2010; Crane et al., 2013; De Felice, 2013; De Felice and Lourenco, 2015; Chatterjee et al., 2016). Because AD has been considered a disease of memory, studies on AD pathogenesis have mainly concentrated on how memory and cognitive failure develop, while other symptoms and co-morbidities have remained largely overlooked.

**Figure 1:**
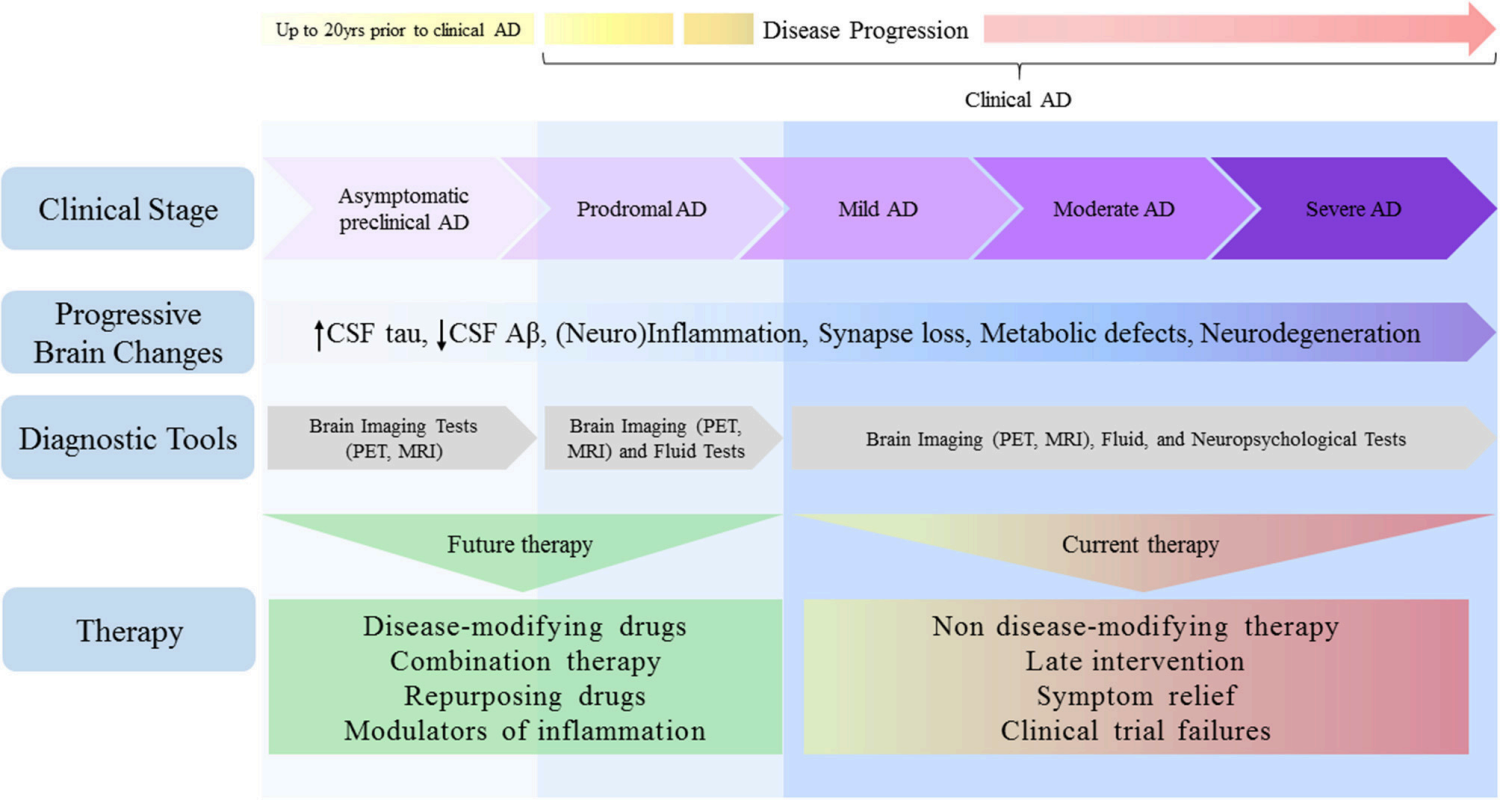
Alzheimer's disease depicted as a continuum: challenges for therapy. Pathophysiological changes in the AD brain begin many years prior to clinical manifestations of disease and move along a continuum, spanning from clinically asymptomatic to severely impaired spectra. Although cognitive symptoms are absent in the preclinical stage, progressive amyloid deposition could drive the patient toward prodromal AD stage, characterized by short-term memory impairment without affecting activity of daily living. As disease progresses, however, many brain areas and their functions become impaired, culminating in severe memory loss and metabolic derangements, both of which affect autonomy. Despite the lack of *bona fide* biomarkers to date, earlier detection will ensure that treatments reach individuals in a timely manner. Given that therapies initially considered promising have disappointed in clinical trials, current AD research pipeline requires a shift toward the use of disease-modifying approaches, combination and/or repurposing therapies, and the search for agents selectively targeting specific modulators of inflammation.

Thus, it is not surprisingly that precise and reliable biomarkers are still lacking for early disease diagnosis. Although conclusive diagnostics has mostly been confirmed through *post-mortem* examination, it is now widely accepted that pathophysiological changes begin to develop decades prior to initial cognitive symptoms, in a preclinical or presymptomatic stage (Sperling et al., 2011a,b). Further, the addition of novel biomarkers to diagnostic criteria has prompted a shift in how AD is considered as pathological entity, increasing the appreciation that it should not be regarded as having discrete and defined clinical stages, but rather as multifaceted process moving along a continuum (Sperling et al., 2011a; van Maurik et al., 2017; Figure 1). Relatively accurate diagnosis and timely therapies will likely be achieved when neuropsychological, fluid and imaging biomarkers are used in combination (Viola and Klein, 2015; Dubois et al., 2016; Blennow, 2017).

Although advances in animal and clinical research over the past few decades have improved our knowledge on the pathophysiological course of AD, even drugs with successful preclinical assessment have not been effective in reversing or slowing down AD progression in large clinical trials. These constraints may be due to that clinical trials have predominantly focused on therapies based on anti-amyloid strategies, since the amyloid cascade hypothesis has been placed at the center of therapeutic prospection (Karran et al., 2011; Cummings et al., 2014; Hendrix et al., 2016). Such disappointing outcomes are also suggestive of problems in translating therapies from rodent model species to humans (De Felice and Munoz, 2016). The lack of adequate control for sex differences in animal models adds up to this translational impedance. Therefore, potential therapies that work in a sex of one animal species (usually male rodents) frequently fail to translate to human trials dominated by female participants (often 2:1 female:male in large trials). Furthermore, while neuropathological features of AD are widely recognized, the intricacies of the mechanism involving central and peripheral derangements have not been clearly defined.

Given that AD holds a complex pathology, it has now been believed that more effective treatments could be possible using disease-modifying therapies and drugs targeting multiple molecular pathways (Castellani and Perry, 2012; Cummings et al., 2014; Perry et al., 2014; Stephenson et al., 2014). These should importantly take sex differences into consideration, as recently noticed (Snyder et al., 2016; Zhao et al., 2016). In this review, we discuss recent advances in the AD field, as well as classical and novel mechanisms that might reveal potential new strategies to treat AD.

## Molecular pathogenesis of AD

### Tau phosphorylation, amyloid deposition, and Aβ oligomers

The most distinctive features present in memory-associated brain regions of AD patients are the intracellular neurofibrillary tangles (NFTs) and the extracellular amyloid plaques. The major component of the NFTs is abnormally phosphorylated and aggregated tau protein (Querfurth and LaFerla, 2010; Medeiros et al., 2011; Morris et al., 2011), thereby destabilizing microtubules and compromising axonal transport (Querfurth and LaFerla, 2010; Ittner and Götz, 2011; Medeiros et al., 2011; Morris et al., 2011; Scheltens et al., 2016). It has been recently shown that tangles induce neuronal loss and spatial memory defects (Fu et al., 2017), putatively providing a link between tau pathology and cognitive deficits in early AD. Although pathological alterations of tau were thought to be downstream events of Aβ deposition, it is equally plausible that tau and Aβ act in parallel to enhancing each other's toxic effects and initiate the pathogenic events germane to AD (Small and Duff, 2008; Spires-Jones and Hyman, 2014; Bennett et al., 2017). Fresh evidence has also pointed to soluble, diffusible tau oligomers as important drivers of synaptotoxicity, and possible culprits for the marked progression of tau pathology across the brain (Fá et al., 2016; Carrieri et al., 2017; Piacentini et al., 2017; Puzzo et al., 2017; Reilly et al., 2017).

The amyloid cascade hypothesis suggests that brain accumulation of the amyloid-β peptide (Aβ), produced by sequential cleavage of the amyloid precursor protein (APP) by the β- and γ-secretase enzymes, is a central event in AD (Karran et al., 2011; Selkoe and Hardy, 2016). Soluble Aβ undergoes conformational changes to high β-sheet content, rendering it prone to aggregation into polymeric forms, including soluble oligomers and larger insoluble fibrils. These fibrils ultimately deposit into extracellular amyloid plaques in the AD brains (Stine et al., 2003; Blennow et al., 2006; Finder and Glockshuber, 2007; Lee et al., 2007).

Aβ is physiologically degraded by the peptidases insulin-degrading enzyme, neprilysin, and by endothelin-converting enzyme (Qiu et al., 1998; Iwata et al., 2001; Farris et al., 2003; Leissring et al., 2003). In addition, Aβ can be cleared out by transportation to peripheral circulation across multiple pathways, including the blood-brain barrier, interstitial fluid bulk flow, arachnoid villi, and glymphatic-lymphatic pathways (Tarasoff-Conway et al., 2015). Additionally, Aβ aggregates can be phagocited and degraded by microglia, perivascular macrophages, and astrocytes. Defective clearing systems could thus lead to an imbalance between production and clearance of Aβ in the brain, thereby resulting in subsequent neuronal dysfunction and neurodegeneration (Hardy, 2002).

A growing body of evidence indicates, however, that plaque deposition is not the sole responsible for the impairments observed in AD. On the other hand, the notion that Aβ oligomers (AβOs) are the main toxins responsible for synapse dysfunction and cognitive deficits in AD has attracted considerable attention to improve our understanding of the mechanisms of the disease (Walsh and Selkoe, 2007; Selkoe, 2008; Ferreira and Klein, 2011; Ferreira et al., 2015; Yang et al., 2017). In this context, plaques have been thought to comprise a reservoir from which AβOs diffuse, or may even act sequestering soluble oligomers until they reach a physiological plateau (Selkoe and Hardy, 2016).

A considerable number of studies has reported that AβOs accumulate in the brain and CSF of AD patients (Georganopoulou et al., 2005; Haes et al., 2005; Anker et al., 2009; Xia et al., 2009; Herskovits et al., 2013; Viola et al., 2014; Murakami et al., 2016), and are found in association with synapses in the brains of patients presenting clinical signals of dementia (Koffie et al., 2009; Bjorklund et al., 2012; Perez-Nievas et al., 2013; Bilousova et al., 2016), adding clinical relevance to their role in AD. These studies suggest that synapse-associated AβOs promote detrimental modifications in synapse structure and composition, thereby leading to memory loss. This growing body of evidence props up an early notion that cognitive decline is not only a result of the extracellular accumulation of Aβ and intracellular accumulation of tau but also as a consequence of synapse failure and loss in AD (Terry et al., 1991; Masliah et al., 1992; Selkoe, 2002).

Despite intense research, the exact mechanisms of how AβOs exert their toxicity remains to be fully unveiled. Binding of Aβ aggregates to various receptors may disrupt key neuronal functions. However, the complete identity of receptors to which they bind and the underlying signaling pathways still remain to be fully elucidated (Ferreira et al., 2015).

We now know that AβOs bind to cell surface receptors and trigger multiple aberrant signaling pathways, including calcium signaling (Mattson, 2010; Ferreira et al., 2015), oxidative stress (Smith et al., 1998; Perry et al., 2002; De Felice et al., 2007), derangements in plasticity-related receptors and increased glutamate release from pre-synaptic terminals (Roselli et al., 2005; Shankar et al., 2007; Decker et al., 2010a; Ferreira et al., 2015). In addition, they promote tau hyperphosphorylation (De Felice et al., 2008; Jin et al., 2011), impaired axonal transport (Snyder et al., 2005; Decker et al., 2010b; Miñano-Molina et al., 2011; Bomfim et al., 2012), and drive inhibition of long-term potentiation (LTP) and memory impairment (Rowan et al., 2005; Shankar et al., 2008; Ferreira and Klein, 2011; Ferreira et al., 2015; Yang et al., 2017).

### Inflammatory markers in the brain

AD pathogenesis appears to include strong interactions with immune mechanisms in the brain. AβOs induce aberrant reactivity of astrocytes and microglia, in the brains of mice and non-human primates (Bomfim et al., 2012; Ledo et al., 2013, 2016; Forny-Germano et al., 2014). Recent studies have further unveiled that disturbances in microglia, as well as interactions with peripheral immune cells, may play key roles in causing synapse loss and neurodegeneration in AD (Browne et al., 2013; Zhang et al., 2013; Baruch et al., 2015, 2016; Guillot-Sestier et al., 2015; Zenaro et al., 2015; Hong et al., 2016a,b). These studies are in line with emerging evidence suggesting that inflammation has a pivotal role in disease pathogenesis, as markers of inflammation, such as TNF-α, IL-1β, IL-6, and other cytokines, have been shown to be increased in the brain, CSF, and plasma of AD patients (Perry et al., 2010; Swardfager et al., 2010; Czirr and Wyss-Coray, 2012; Alcolea et al., 2014; Heneka et al., 2015a; Hong et al., 2016a; Salter and Stevens, 2017).

Increased pro-inflammatory signaling resulting from reactive microglial reduces Aβ clearance, promotes aberrant synaptic pruning (Lee and Landreth, 2010; Mandrekar-Colucci et al., 2012; Heneka et al., 2015a,b; Hong et al., 2016b), prompts Aβ and tau pathologies, and contributes to impaired synapse function (Wang W. Y. et al., 2015). Importantly, TNF-α-dependent mechanisms appear to drive memory defects (Lourenco et al., 2013) and depressive-like behavior in AD mice (Ledo et al., 2016), thereby indicating a causal role of inflammation in deleterious processes linked to AD.

### Unfolded protein response and defective proteostasis

Pro-inflammatory pathways triggered by AβOs, notably via TNF-α, have been reported to induce neuronal stress (Lourenco et al., 2013), likely resulting in defective proteostasis. Furthermore, it has been recently demonstrated that AβOs stimulates eIF2α phosphorylation (Devi and Ohno, 2010, 2013, 2014; Lourenco et al., 2013; Ma et al., 2013; Baleriola et al., 2014). In the brain, eIF2α is a hub that controls protein synthesis-dependent learning and memory and mantain neuronal integrity in health and disease. When phosphorylated, however, eIF2α attenuates the initiation of global protein synthesis (Lourenco et al., 2015).

Aberrant eIF2α phosphorylation and inhibition of protein synthesis have emerged as major molecular pathways driving synapse and memory failure in AD models (Costa-Mattioli et al., 2007; Lourenco et al., 2013, 2015; Ma et al., 2013; Baleriola et al., 2014). In line with this notion, deletion of eIF2α kinases, including PKR, PERK, or GCN2 restores memory and synapse function in mouse models of AD (Lourenco et al., 2013; Ma et al., 2013).

Abnormal accumulation of misfolded proteins in the endoplasmic reticulum triggers the unfolded protein response (UPR), a set of signaling branches aimed at restore cellular homeostasis (Hetz, 2012; Dufey et al., 2014; Hetz and Saxena, 2017). However, when prolonged, UPR signaling might compromise neuronal functions, resulting in neurodegeneration (Lourenco et al., 2015; Freeman and Mallucci, 2016; Hetz and Saxena, 2017). There is now considerable evidence suggesting that AD brain display increased markers of UPR (Hoozemans et al., 2009; Hetz and Saxena, 2017), and that at least the PERK (Ma et al., 2013) and IRE-1a (Lourenco et al., 2013; Duran-Aniotz et al., 2017) branches of UPR are involved in memory defects in AD mice. Further, the chemical chaperone 4-phenylbutyrate alleviates AβO-induced memory defects in mice (Lourenco et al., 2013), thus highlighting the role of UPR in mediating neurotoxicity in AD. The combination of misfolded protein accumulation, activation of brain immune responses and defective proteostasis might thus comprise the very essence of synapse and memory failure in AD.

## Novel pathophysiological mechanisms in AD

Scientific breakthroughs during the past decades have expanded our knowledge on cellular and molecular aspects of AD. Nevertheless, AD remains largely idiopathic, and therapies that effectively combat disease progression are still lacking. Given that AD largely associates with memory loss, it is not surprising that the vast majority of studies deal with mechanisms implicated in cognitive deterioration. Hence, much less is known about how brain regions that are not directly linked to memory are affected in AD, as well as about mechanisms underlying its major comorbidities.

Numerous studies have investigated how Aβ impacts the hippocampus and the cortex (Ferreira and Klein, 2011; Musiek and Holtzman, 2015), known to be fundamentally involved in acquisition, consolidation, and recollection of new episodic memories. However, early studies indicated that brain regions not necessarily involved in learning and memory might also be affected in AD. It is noteworthy that AD patients exhibit significant non-cognitive deficits (summarized in the Table 1) such as sleep-wake disorders and neuroendocrine alterations attributable to hypothalamic dysfunction (Prinz et al., 1982; White et al., 1996; Csernansky et al., 2006).

**Table 1:** Novel pathophysiological mechanisms in AD.

**Feature**	**Pathophysiological mechanisms**	**References**
Impaired hypothalamic function	– Aβ deposits in hypothalamic nuclei leading to disturbances in circadian rhythm; – Reduced dendrite arborization and neurodegeneration; – Inflammation driving endoplasmic reticulum stress and insulin resistance.	Ogomori et al., 1989; Standaert et al., 1991; Duncan et al., 2012; Lim et al., 2014; Baloyannis et al., 2015; Clarke et al., 2015; Musiek et al., 2015; Musiek and Holtzman, 2016; Kincheski et al., 2017; Stevanovic et al., 2017
Metabolic derangement	– Reduced cerebral glucose metabolism; – Altered peripheral metabolism with hyperglycemia and hyperinsulinemia; – Defective glucose metabolism and insulin signaling induced by Aβ;	Chase et al., 1984; Janson et al., 2004; Rivera et al., 2005; Steen et al., 2005; Lester-Coll et al., 2006; De Felice et al., 2009; de la Monte, 2009; Matsuzaki et al., 2010; Moloney et al., 2010; Bomfim et al., 2012; Talbot et al., 2012; Crane et al., 2013; De Felice, 2013; Lourenco et al., 2013; De Felice and Ferreira, 2014; Clarke et al., 2015
Disturbances in monoamine signaling and mood	– Aβ induces both depressive-like behavior and decreases brain serotonin levels; – Increased microglial activity of IDO might partially explain reduced serotonin levels; – Reduced tryptophan and increased quinolinic acid in plasma might drive depressive-like behavior in AD; – Alterations in the dopaminergic system, including reduced levels of dopamine and its receptors might contribute to hippocampus-dependent memory deficits and reward circuitry dysfunction.	Gibb et al., 1989; Storga et al., 1996; Burns et al., 2005; Bonda et al., 2010; Gulaj et al., 2010; Jürgensen et al., 2011; Ledo et al., 2013, 2016; Romano et al., 2014; Masters et al., 2015; Nobili et al., 2017
Inflammation	– Pro-inflammatory cytokines are elevated in AD brains and mediate neurotoxic signals; – Brain inflammation underlies defective neuronal insulin signaling and peripheral metabolic deregulation; – Inflammation may drive synaptic failure in the monoaminergic systems, thereby linking cognitive and non-cognitive symptoms found in AD patients.	Heneka and O'Banion, 2007; Bonda et al., 2010; Lee and Landreth, 2010; Swardfager et al., 2010; Bomfim et al., 2012; Czirr and Wyss-Coray, 2012; Ledo et al., 2013, 2016; Lourenco et al., 2013; Alcolea et al., 2014; De Felice and Ferreira, 2014; Morales et al., 2014; Clarke et al., 2015; Heneka et al., 2015a,b; Yirmiya et al., 2015; Hong et al., 2016a,a; Santos et al., 2016; Nobili et al., 2017; Salter and Stevens, 2017

*Aβ, amyloid-β peptide; AD, Alzheimer's disease; CNS, central nervous system; IDO, indolamine-2,3-dioxygenase; T2D, type 2 diabetes*.

### Impaired hypothalamic function

Disturbances in hypothalamic nuclei have been reported in patients and animal models of AD (Duncan et al., 2012; Lim et al., 2014; Musiek et al., 2015; Musiek and Holtzman, 2016; Stevanovic et al., 2017). Since the hypothalamus is responsible for controlling circadian rhythm, impairments in its function can at least partially account for sleep disturbances. Nonetheless, although initial results have already shed light on how sleep becomes deregulated in AD (Ju et al., 2014; Musiek and Holtzman, 2016; Kincheski et al., 2017), studies investigating whether hypothalamic defects mediate sleep disturbances in AD are still needed.

Derangements in hypothalamic functions play a central role in peripheral metabolism deregulation and its consequences. For instance, hypothalamic inflammation and impaired proteostasis are critical pathogenic events in the establishment of peripheral insulin resistance in metabolic disorders (Zhang et al., 2008; Milanski et al., 2009; Denis et al., 2010; Arruda et al., 2011; Thaler et al., 2012; Valdearcos et al., 2015). Nonetheless, very few studies so far investigated hypothalamic dysfunction in AD.

Early *post-mortem* studies identified Aβ deposits in hypothalamic nuclei of AD patients (Ogomori et al., 1989; Standaert et al., 1991), and neurodegeneration with marked retraction of dendrites in early AD (Baloyannis et al., 2015). Further, hypothalamic endoplasmic reticulum stress, inflammation, and insulin resistance were demonstrated in AβO-injected mice and non-human primates (Clarke et al., 2015). Dysfunction triggered by AβOs in the hypothalamus associated with development of persistent peripheral glucose intolerance, which was further demonstrated in several transgenic mouse models of AD (Clarke et al., 2015; Vandal et al., 2015; Stanley et al., 2016), and in human patients (Craft et al., 1992).

### Defective glucose metabolism and insulin signaling

Altered peripheral metabolism with hyperglycemia and hyperinsulinemia, which are cardinal features of type 2 diabetes (T2D), were recently found to positively correlate with development of AD-like brain pathology in humans (Matsuzaki et al., 2010; Crane et al., 2013). Conversely, AD has been associated with increased T2D risk (Janson et al., 2004), suggesting that the connection between AD and T2D may comprise a two-way road. AD progression positively further correlates with reduction of cerebral glucose metabolism in the forebrain, including the posterior parietal lobe and portions of temporal and occipital lobes (Chase et al., 1984).

An important player accounting for impaired glucose metabolism in AD could arise from defects in insulin signaling pathways. AD brains exhibit lower levels of insulin and reduced insulin receptor (IR) expression and sensitivity (Rivera et al., 2005; Steen et al., 2005; Talbot et al., 2012). Further, impairments in insulin signaling downstream machinery have been reported in *post-mortem* brain tissue and in animal models of AD (Steen et al., 2005; Lester-Coll et al., 2006; de la Monte, 2009; Moloney et al., 2010; Bomfim et al., 2012; Craft, 2012; Talbot et al., 2012; Lourenco et al., 2013; Clarke et al., 2015). Recent studies have shown that AβOs are the toxins linked to impaired hippocampal insulin signaling by promoting internalization and cellular redistribution of insulin receptors, blocking downstream hippocampal insulin signaling (De Felice et al., 2009; Ma et al., 2009; Bomfim et al., 2012). Such body of evidence has established novel molecular parallels between AD and T2D.

The precise molecular mechanisms connecting impaired glucose metabolism and insulin signaling to AD pathogenesis remain to be fully determined. Nonetheless, mounting evidence has pointed to inflammation as a critical player linking AD and metabolic diseases, including T2D (De Felice and Ferreira, 2014; Ferreira et al., 2014; Morales et al., 2014; Heneka et al., 2015b). Overproduction of pro-inflammatory cytokines, notably TNF-α, is a key feature of the pathophysiology of metabolic disorders (Hotamisligil, 2006, 2017). Notably, brain inflammation has recently been proposed to underlie defective neuronal insulin signaling (Bomfim et al., 2012; Lourenco et al., 2013), as well as peripheral metabolic deregulation in AD (Clarke et al., 2015).

### Disturbances in monoamine signaling and mood

Mounting evidence supports the notion that microglial activation and brain inflammation could further underlie mood disorders, including depressive behaviors (Yirmiya et al., 2015; Santos et al., 2016). Depression and/or apathy have been reported as frequent comorbidities in AD patients (Lyketsos and Olin, 2002), and have been regarded as risk factors for AD (Green et al., 2003; Ownby et al., 2006; Starkstein and Mizrahi, 2006; Geerlings et al., 2008).

Although clinical and epidemiological studies have revealed a strong connection between AD and depression, the mechanisms connecting these disorders at the molecular and cellular levels have only recently begun to be established. Clues into a mechanistic link between memory and mood disturbances in AD came from recent works showing that AβOs induce both depressive-like behavior and memory deficits in mice and associate with decreased brain serotonin levels (Ledo et al., 2013, 2016) in a similar way to that observed in transgenic mice model of AD (Romano et al., 2014). Reduced serotonin levels may be linked to increased levels and activity of indolamine-2,3-dioxygenase (IDO) follow microglial activation. Interestingly, AD patients were found to have reduced levels of plasma tryptophan and increased quinolinic acid (Gulaj et al., 2010), as well as increased IDO immunoreactivity in microglia (Bonda et al., 2010). Because inflammation plays a significant role in depression, these findings raise the possibility that AβO-induced brain inflammation may constitute a common denominator between cognitive and mood alterations in AD.

Alterations in the dopaminergic system have also been reported in AD patients and experimental models, including reduced levels of dopamine and its receptors (Gibb et al., 1989; Storga et al., 1996; Burns et al., 2005; Jürgensen et al., 2011; Nobili et al., 2017), and are commonly linked to cognitive and non-cognitive symptoms of the disease. It has been recently shown that inflammation and apoptosis take place in the ventral tegmental area, causing selective degeneration of the dopaminergic nuclei before senile plaque deposition, tangles or any sign of neuronal loss in cortical and hippocampal regions in a transgenic mouse model of AD (Nobili et al., 2017).

Given that dopaminergic neurons from ventral tegmental area not only modulate hippocampal synaptic plasticity (Rossato et al., 2009; McNamara et al., 2014; Broussard et al., 2016), but also target the nucleus accumbens and the cerebral cortex (Russo and Nestler, 2013), dopaminergic degeneration in ventral tegmental area might largely contribute to the deficits in hippocampus-dependent memory and reward circuits. These findings may provide an intriguing explanation to recent observations in AD patients indicating that the clinical diagnosis of dementia is associated with early non-cognitive symptoms, such as depression and apathy (Masters et al., 2015). Overall, these recent data suggest that inflammation may drive synaptic failure in the monoaminergic systems, thereby linking the cognitive and non-cognitive symptoms found in AD patients.

## Challenges for AD therapy

Despite intensive investigation of mechanisms of pathogenesis in AD during the past three decades, little has been achieved in terms of effective treatments or approaches to prevent or cure it. Taking into account the dramatic rise in the number of AD cases, huge economic and social hurdle will impact the society if no treatment is developed within the next few years. Additionally, it is noteworthy that advances in therapeutic strategies for AD that lead to even small delays in AD onset or progression would significantly attenuate the global burden of the disease.

Given the conceptual frameshift that occurred in the field in the past few years, AD has not only been viewed with discrete and defined clinical stages, but as a multifaceted process moving along a continuum. Thanks to the evolving biomarker research, it is now recognized that pathophysiological changes begin many years before clinical manifestations of AD. For example, changes in CSF tau levels have been shown to develop ~15 years before the onset of clinical AD, while CSF Aβ42 levels may drop even earlier, up to 20 years before symptom onset (Bateman et al., 2012; Buchhave, 2012; Villemagne et al., 2013; Fagan et al., 2014).

The spectrum of AD spans from clinically asymptomatic to severely impaired (Figure 1). However, these boundaries are challenging, given that separation between healthy aging and preclinical AD is not well-defined in our current understanding. This unmet question will likely be addressed in the future, as early detection biomarkers have become a major research focus.

Sex differences should also be taken into account as a biological variable in AD pathogenesis as women constitute the majority of affected people, accounting for nearly two-thirds of AD patients (Alzheimer's Association, 2017). Reasons for the higher frequency of AD among women could be partly explained by the fact that women live longer. However, late-onset AD risk is greater in women even after controlling for their longer lifespan relative to men (Viña and Lloret, 2010). The biological underpinnings of the increased AD risk in women remain largely unknown.

Nonetheless, it is now accepted that the perimenopause to menopause transition disrupts multiple estrogen-regulated systems, thereby affecting multiple domains of cognitive function (Brinton et al., 2015; Christensen and Pike, 2015). Indeed, recent preclinical studies have implicated that a shift in the bioenergetics system of the brain during menopause onset could serve as an early initiating mechanism for increased AD risk in the female brain (Brinton et al., 2015; Mosconi et al., 2017a,b). These biological variables may lead to increased fatty acid catabolism, Aβ deposition, and impaired synaptic plasticity (Liu et al., 2008; Brinton, 2009; Yao and Brinton, 2012), which could serve as a mechanism that triggers AD (Brinton et al., 2015). As a result, it is conceivable that disappointing outcomes in clinical trials may be partially explained by metabolic differences in women and men. Therefore, recommendations to include both female and male animals in preclinical research should be completely embraced by the research community.

While the amyloid cascade hypothesis has dominated research for the past 20 years, the shift toward disease-modifying drug development in the last decade might be imperative to develop approaches that interrupt the underlying disease processes.

Potential benefits for AD therapy can also emerge from combination pharmacotherapy. This strategy has proven effective for several diseases, including tuberculosis, HIV/AIDS, cardiovascular diseases, and cancer (Perry et al., 2014; Hendrix et al., 2016), and holds potential to enhance the efficacy of drugs that are ineffective on their own, but offer synergistic or additive benefits in combination.

Taking into account the well-known high failure rates in drug development targeting the central nervous system, strategies aimed at repurposing already marketed drugs become an interesting option to speed up drug discovery in AD (Appleby and Cummings, 2013). Given that metabolic derangements seem to play a pivotal role in AD, and that a myriad of drugs for metabolic disease have already been labeled for human use, repurposing such compounds may have the potential to accelerate drug development. That is because preclinical toxicology, human safety, tolerability, and pharmacokinetic assessments could move faster. Impaired brain insulin signaling or brain insulin resistance seems play a central role in the molecular pathogenesis of sporadic AD. Thus, targeting brain insulin signaling through the administration of drugs that have already been previously approved for the treatment of diabetes mellitus, such as insulin and drugs that improve insulin sensitivity, could expedite their development for the treatment of AD (Chen et al., 2016). It worthy to note that anti-diabetic compounds, such as insulin, exenatide, and liraglutide, have already been tested in ongoing clinical trials (clinical trial ID NCT01767909, NCT01255163, and NCT01843075, respectively).

Neuroinflammation, especially at the earliest stages, supports a vicious cycle of microglial activation, release of pro-inflammatory factors, and neuronal damage. Additionally, inflammatory mechanisms, such as those driven by TNF-α, may be orchestrated between the brain and the periphery, providing a likely link between AD and peripheral metabolic deregulation (De Felice and Ferreira, 2014; Ferreira et al., 2014; De Felice and Lourenco, 2015). The important role of neuroinflammation in AD is further supported by findings that gene variants for immune receptors, including TREM2, are associated with altered AD risk (Guerreiro et al., 2013; Heneka et al., 2015a).

A considerable body of evidence supports that inflammation could be a therapeutically relevant target in AD. Nevertheless, trials with anti-inflammatory compounds, such as non-steroidal anti-inflammatory drugs (NSAIDs), peroxisome proliferator-activated receptor-γ (PPAR-γ) activators, minocycline, and TNF-α signaling inhibitors have not yet provided exciting outcomes to date (Calsolaro and Edison, 2016), although lifelong use of NSAIDs has been associated with reduced risk of developing AD (Wang J. et al., 2015).

Additional therapeutic approaches with intravenous immunoglobulins and/or monoclonal antibodies are currently under evaluation, and results have not been conclusive yet. These uncertain results could be, to some extent, due to that anti-inflammatory drugs target generic rather than specific neuroinflammatory components in AD. Thus, specific modulators of inflammation at early disease stages will be essential to understand the potential of targeting inflammation in neurodegeneration.

## Concluding remarks

Although our understanding of AD has considerably increased over recent years, there is a still unmet requirement for effective therapeutics. Properly diagnosing AD is still one of the major hurdles in the field, as reliable biomarkers are lacking. There is fresh and compelling preclinical evidence that brain regions not necessarily involved in learning and memory might also be affected in AD, driving its major comorbidities. As most of therapeutic approaches have had disappointing outcomes so far, it is time to revisit the science underlining our current AD canons, and move toward the search for additional disease mechanisms and keys to treatment. Inflammation plays a critical role in the pathogenesis of AD and seems to drive the metabolic derangements that have been found to positively correlate with disease onset, leading to the emergence of cognitive and non-cognitive symptoms.

A deeper understanding of the complex features underlying major disease symptoms, including behavioral, mood, inflammation, and metabolic disturbances, may contribute to the development of novel and successful therapies. Given the differential prevalence of AD in men and women, sex differences should also be taken into account when studying AD pathophysiology, as they might reveal the need for separate therapeutic approaches. Drugs currently approved for use in AD are not disease-modifying, only confer mild and transient symptomatic management. Intervention at earlier stages using disease-modifying and combination therapy comprised of repourposed drugs and anti-inflammatory agents could pave the road toward successful outcomes in AD therapy.

## Author contributions

RF, ML, and FD: planned, researched and wrote the manuscript.

### Conflict of interest statement

The authors declare that the research was conducted in the absence of any commercial or financial relationships that could be construed as a potential conflict of interest.
